# Current Trends in Mycotoxin Detection with Various Types of Biosensors

**DOI:** 10.3390/toxins15110645

**Published:** 2023-11-06

**Authors:** Krisztina Majer-Baranyi, Nóra Adányi, András Székács

**Affiliations:** 1 Food Science Research Group, Institute of Food Science and Technology, Hungarian University of Agriculture and Life Sciences, Villányi út 29-43, H-1118 Budapest, Hungary; baranyi.krisztina@outlook.com; 2 Agro-Environmental Research Centre, Institute of Environmental Sciences, Hungarian University of Agriculture and Life Sciences, Herman Ottó út 15, H-1022 Budapest, Hungary; szekacs.andras@uni-mate.hu

**Keywords:** food safety, mycotoxin, antibody, molecularly imprinted polymers, aptamers

## Abstract

One of the most important tasks in food safety is to properly manage the investigation of mycotoxin contamination in agricultural products and foods made from them, as well as to prevent its occurrence. Monitoring requires a wide range of analytical methods, from expensive analytical procedures with high-tech instrumentation to significantly cheaper biosensor developments or even single-use assays suitable for on-site monitoring. This review provides a summary of the development directions over approximately a decade and a half, grouped according to the biologically sensitive components used. We provide an overview of the use of antibodies, molecularly imprinted polymers, and aptamers, as well as the diversity of biosensors and their applications within the food industry. We also mention the possibility of determining multiple toxins side by side, which would significantly reduce the time required for the analyses.

## 1. Introduction

The source of contamination of food and environmental samples can be of both chemical and biological origin. When detecting chemical pollutants, one of the most common tasks is examining chemical residues. Researchers have developed fast methods to be utilised in procedures for the simultaneous determination of specific groups of compounds, such as various types of plant protection agents and veterinary drugs. Mycotoxins are also considered chemical pollutants: their emergence indicates biological pollution and the presence of toxinogenic, and often pathogenic, fungi. Humans suffer internal exposure to mycotoxins directly through the consumption of infected foods, such as peanuts, nuts, cereals, beans, or oilseeds. Certain mycotoxins also appear as emerging contaminants in surface waters; therefore, limited exposure may become apparent through drinking water. Indirect exposure can occur through animals that consume contaminated feed, primarily affecting products like milk, eggs, and offal. Considering the most important mould species and their toxins, certain economic crops such as cereals (especially corn), hot peppers, certain fruits (like apples and grapes), and products derived from them may become infected. In the European Union (EU), the Rapid Alert System for Food and Feed (RASFF), operating in EU member states, identified certain nuts (like pistachios and peanuts), along with coffee and cocoa beans, as well as spices in imported products, as the primary sources of mycotoxin exposure.

Mycotoxins are primarily produced by various filamentous fungi, with the most important ones being *Aspergillus*, *Penicillium*, *Alternaria*, and *Fusarium* species. Additionally, *Claviceps* and *Stachybotrys* species are also significant producers of mycotoxins. Mycotoxins are small-molecular-weight toxic secondary metabolites produced by specific fungal species. They form a group of compounds with diverse chemical structures [1]. The current number of identified mycotoxins is approximately 300–400 [2,3]. Considering their appearance in feed and food, as well as their health effects, only a few groups of mycotoxins are considered hazardous. These include aflatoxins (AFs), fumonisins (FMs), ochratoxins (OTs), trichothecenes, zearalenone (ZON), and their derivatives. These major mycotoxins routinely occur in a wide range of food and feed products of plant and animal origin. While of lesser importance, patulin (PAT) and citrinin (CIT) mainly contaminate fruits, fruit juices, and cereals [1]. Another mycotoxin that occurs in cereal and fruits is alternariol (AOH). The presence of fungi adversely affects the quality of food, food raw materials, and feed. They significantly contribute to the deterioration of the sensory properties, the reduction in nutritional value, and in the health-damaging effects of the mycotoxins they produce.

AFs are primarily produced by *Aspergillus flavus* and *A. parasiticus*. The most significant naturally occurring AFs are aflatoxin B1, B2, G1, and G2 (AFB1, AFB2, AFG1, and AFG2). These compounds are chemically multi-conjugated, polycyclic molecules with a benzpyrene structure. The hydroxylated metabolites M1 and M2 (AFM1 and AFM2) are secreted in milk. AFs are known to cause liver damage, and they are genotoxic and immunosuppressive. Their toxicity order is as follows: AFB1 > AFG1 > AFB2 > AFG2. These toxins are most commonly found in peanuts, but they can also be present in other foods such as soy, rice, millet, corn, oats, beans, coffee, and peppers, as well as in different types of feed. They are particularly prevalent in regions with warm climates and during rainy, high-humidity weather. However, a series of comprehensive studies indicate that AFs have recently emerged in South-East European countries due to climate change [4,5]. A less frequently occurring but structurally closely related mycotoxin is sterigmatocystin (STC), the polyketide penultimate precursor of AFB1 and AFG1. Its appearance can be expected when cereals and/or foods are contaminated with fungi that produce AFs. This mycotoxin damages cereals, and animals can also become infected through it, causing significant economic damage to the biotechnology, agricultural, and food industries [6].

Ots are produced by *Aspergillus* and *Penicillium* species and are most often found in cereals, legumes, cocoa, coffee, soybeans, rice, wine, dried fruits, and spices. Based on their chemical structure, they are β-phenylalanine derivatives related to dihydrocoumarin. There are two main representatives known as ochratoxins A and B (OTA and OTB), and their ethyl and methyl ester derivatives. OTA is a chlorinated derivative, making it highly toxic. It can cause kidney damage, cancer, immunosuppression, nervous system problems, breathing difficulties, and teratogenic effects. OTA also has a long half-life and is slow to be eliminated from the body. OTs can form in continental climatic conditions as storage moulds produce them in feed due to poor storage practices. Furthermore, toxin formation can occur across a wide temperature range (between 4 and 30 °C), and their concentration can become quite high in certain areas [7].

Mycotoxins produced by different strains of *Fusarium* are trichothecenes (deoxynivalenol, or DON, is the main representative), T-2 toxin (T-2), HT-2, FMs (e.g., fumonisin B1, or FB1), and ZON. After Afs, *Fusarium*-derived toxins are the most frequently occurring mycotoxins in agricultural products, and, if they enter the food and feed chain, they can exert a variety of harmful effects on human and animal health [8].

Among ZON derivatives produced by *Fusarium* fungal strains, ZON is the most significant as a natural contaminant of plant products. In terms of its chemical structure, ZON is a resorcinolactone toxin. *Fusarium* species attack cereals and corn while these crops are still in the fields, or occasionally during storage of harvested crops. The growth of the fungus, along with its toxin formation, can continue also due to improper post-harvest management. ZON typically appears as a surface contamination on cereals. After processing, in the mill industry, it can enter the bran [9]. ZON is known to exert estrogenic effects in mammals, leading to the enlargement of the uterus and mammary glands, increased secretion, irregular estrus, abortion, and disruption of sperm production in pigs [10]. However, its carcinogenic effect has not been proven.

Deoxynivalenol (DON), also known as vomitoxin B-type trichothecene compound, is an epoxyterpenoid derivative. DON is primarily found in grains such as wheat, barley, oats, rye, and corn. It causes grain head blight in wheat and ear rot in corn. DON is mainly produced by the fungi *Fusarium graminearum* and *F. culmorum* [9,11,12,13]. The acute effects of exposure to DON include nausea, vomiting, diarrhoea, abdominal and headache pain, and fever. Digestive problems, vomiting, and reduced weight gain can also be observed in pigs due to exposure to DON [8,10,14,15,16].

FB1 produced by *Fusarium* fungi (e.g., *F. verticillioides*, *F. proliferatum*, *F. nygamai*, *F. fujikuroi*, and *F. oxysporum*) is one of the most hazardous mycotoxins, considered potentially carcinogenic. The latest studies have shown that FB1 can also damage the lungs, cerebellum, and kidneys. Its toxicity can promote bacterial infections and even affect the development of autism. FB1 can be found in a wide range of agricultural products, endangering cereals, dried fruits, wine, milk, coffee beans, cocoa, and meat products [17,18]. PAT is produced by several species of *Penicillium* and *Aspergillus*, most importantly *P. expansum*, causing the emergence of PAT in apples and their products, pears, apricots, peaches, and grapes [19,20].

CIT is produced as a secondary metabolite by various species of *Aspergillus*, *Penicillium*, and *Monascus*. CIT can contaminate a wide range of food grains and feed at any stage, including pre-harvest, harvest, drying, and storage. It is commonly found in beans, fruit, fruit and vegetable juices, herbs, and spices, as well as in dairy products and red mouldy rice [21].

Alternaria, known as a plant pathogenic fungus found in fruits and vegetables, produces numerous metabolites during growth and reproduction. Not only are these secondary metabolites considered hazardous, but their spores are also known allergens that can proliferate indoors and cause hay fever or hypersensitivity reactions. The four most representative metabolites produced by fungi of the *Alternaria* genus are AOH, alternariol monomethyl ether (AME), tenuazonic acid (TeA), and tentoxin. These metabolites are usually carcinogenic, mutagenic, and cytotoxic, making them harmful not only to plants but also to the humans and animals that consume them. In addition, AOH is also an androgen agonist mycoestrogen. Since there are no globally established standards and norms for *Alternaria* mycotoxins, their investigation and determination are of utmost importance [19,22,23,24].

Decades ago, the European Commission established the permissible limit of mycotoxins in food. The acceptable AFB1 content of goods intended for direct human consumption or use is 2 mg/kg, the total AF contamination is 4 mg/kg, the maximum allowable amount of OTs is 5–50 mg/kg, while the ZON content can range from 30 to 1000 mg/kg [25]. As per these guidelines, the identification and quantification of mycotoxins require increasingly accurate analytical methods with progressively lower limits of detection (LODs) and limits of quantification (LOQs) [26]. Numerous analytical procedures have been developed for the investigation of mycotoxins [27]. Among the tests, thin-layer chromatography [28], overpressured layer chromatography [29], capillary electrophoresis [30], and high-performance liquid chromatography (HPLC), often with mass spectrometric detection (HPLC-MS) procedures, are the most common. However, the use of biosensors is increasingly gaining prominence in this field.

According to the definition recommended by the International Union of Pure and Applied Chemistry [31], biosensors are “self-contained integrated devices, which are capable of providing specific quantitative or semi-quantitative analytical information using a biological recognition element (biochemical receptor), which is retained in direct spatial contact with an electrochemical transduction element”. Based on its structure, it must be distinguished from analytical measuring instruments, and due to its repeated use, it must be separated from single-use tests and devices. In the process of measuring with a biosensor, the sample is first brought into contact with the biosensing receptor surface. The sensor detects any physical or biochemical changes occurring during the interaction between the analyte and the receptor. The received signal is then converted, stored, and evaluated electronically [32,33]. Detection methods include a wide range of techniques such as fluorescence polarisation, chemiluminescence, surface plasmon resonance (SPR), fluorescence and chemiluminescence resonance energy transfer (FRET and CRET), surface-enhanced Raman scattering (SERS), optical waveguide lightmode spectroscopy (OWLS), quartz crystal microbalances (QCMs), different types of electrochemical (EC) sensors, lateral flow, and nanoarray devices. The operation and efficiency of the sensor are determined by two main components: the specific recognition part and the signal conversion unit. Together, these two components are responsible for the selectivity and sensitivity of the sensor. Countless combinations can be imagined, a great deal of variations of which have been tested in the last nearly fifty years. High sensitivity, low LOD and LOQ, specificity, reproducibility, and robustness are just some of the properties expected of biosensors.

**Figure 1:**
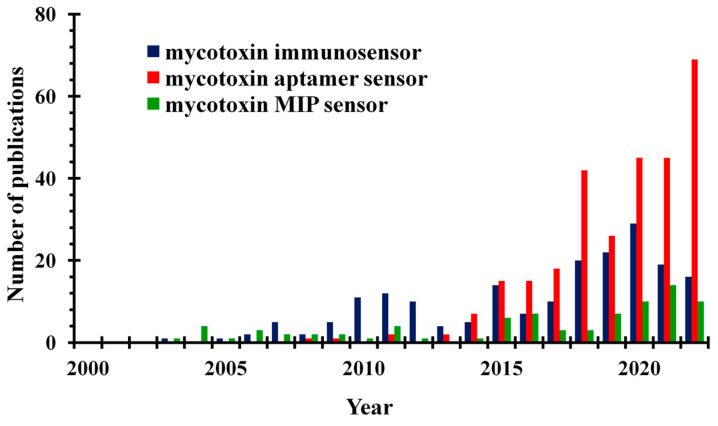
The number of papers published annually during the past two decades using the keywords “mycotoxin immunosensor”, “mycotoxin aptamer sensor”, and “mycotoxin MIP sensor” from the Web of Science database (accessed on 19 April 2023).

The design of the first reported biosensor was based on an immobilised enzyme glucose oxidase, and detection was achieved using an oxygen electrode recording the loss of oxygen [34]. While initially only enzymes were used in biosensors as biologically sensitive substances, later antibodies, organelles, and whole cells were also applied. Nowadays, many researchers report on the operation of sensors prepared using nucleic acids and aptamers (Apts), as well as molecularly imprinted polymers (MIPs) and other molecularly imprinted optosensing materials (MIOMs). Examining publications from the last two decades (Figure 1), it is evident that, in the early 2000s, mainly antibody-based immunosensors were developed for investigating mycotoxins. However, since 2015, the interest in aptasensors has significantly increased. Currently, more researchers are reporting the use of aptamers, while the utilisation of antibodies is declining and MIPs have not gained as much popularity. Several comprehensive reviews concerning mycotoxin analysis using various types of biosensors have been recently published. Badie Bostan et al. [35] summarised advancements in OTA detection via aptasensors, Jiang et al. [36] discussed current progress regarding nanomaterial-based biosensors for OTA determination, while Goud et al. [37] summarised recent developments in mycotoxin detection using nanomaterial-based EC biosensors, including the use of nanoparticles (NPs) with different compositions and forms.

Biosensors can also be grouped according to the method used to detect the complex formed during the biochemical reaction [38]. Since the formation of the complex usually does not yield an easily measurable product, some form of labelling is employed for determination, such as radioactive isotope, enzymes, fluorescent or chemiluminescent molecules, and, more recently, magnetic NPs and time-resolved fluorescent microspheres (TRFMs).

Thanks to the development of measuring technology, especially immunosensors, label-free detection options have emerged. In these methods, the immune reaction is not detected using additional chemical or biochemical reactions; rather, the physical changes occurring during the process are captured using highly sensitive measuring techniques [39].

Lately, lateral flow analysis (LFA) biosensors, which are based on chromatography-based sensor formats, have garnered significant interest. Although they do not strictly belong to the classical definition of biosensors, as they are typically single-use strips, they offer excellent potential for rapid on-site ligand detection. LFA biosensors are portable, provide fast analysis turnaround times, are cost-effective, and are easy to use. Various biorecognition elements, primarily Abs and Apts, can be used for the development of strip sensors. A typical strip comprises five elements: a sample pad, a conjugate zone, a nitrocellulose membrane, an absorbent region, and a backing zone. A small amount of a flowing liquid sample progresses through the device leading to the formation of detectable complexes in the analyte-specific strip zone, thus rendering the analytical information directly visible as a colour signal. The read-out of this test, indicated by lines with varying intensities, can be evaluated visually or using a specialised reader [40].

The development of nanomaterials, owing to their high performance and versatile properties, holds great promise for the application of various types of biosensors. They contribute to the advancement of highly sensitive, selective, and straightforward detection procedures [41]. Numerous nanomaterials can serve as immobilisation agents for biomolecules, signal generators, fluorescence quenchers, or for signal amplification. Examples include gold or silver NPs (AuNPs and AgNPs), carbon-based NPs, magnetic NPs, quantum dots (QDs), and emerging nanomaterials, including upconversion NPs (UCNPs) and metal–organic frameworks (MOFs), as well as hybrid nanostructures.

**Figure 2:**
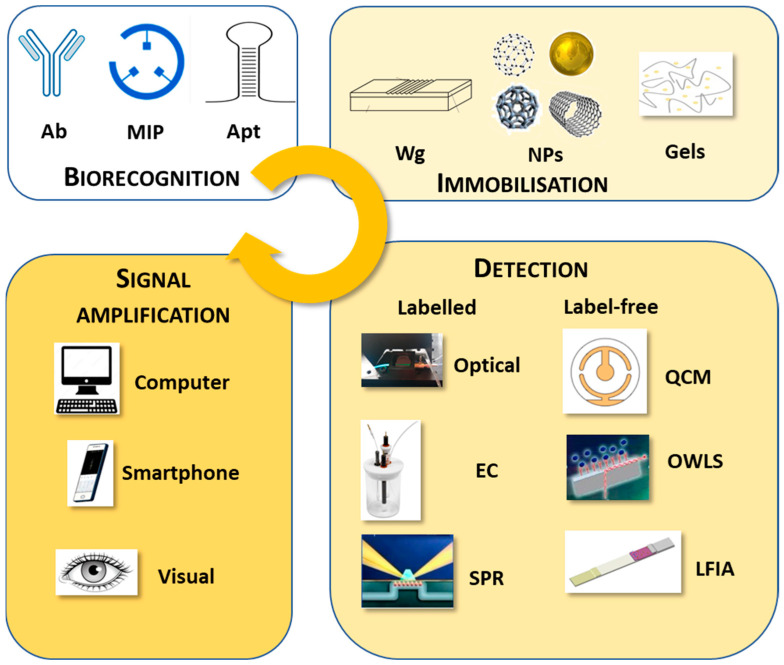
Schematic structures of biosensor setups with their main functional units indicated. Biorecognition: the element providing molecular recognition and specificity—antibodies (Ab), molecularly imprinted polymers (MIP), or aptamers (Apt). Immobilisation: surface type the biorecognition element is anchored to—waveguide (Wg), nanoparticles (NP), or gels (GeI). Detection: method of signal generation—electrochemical (EC), optical (Opt), and lateral flow immunoassay (LFIA), as examples using suitable labels for detection, and optical waveguide lightmode spectroscopy (OWLS), quartz crystal microbalance (QCM), and surface plasmon resonance (SPR), as examples of label-free detection systems. Signal amplification: processing the detection signal into a qualitatively or quantitatively assessable, amplified signal.

In this review, we present the development of sensors designed for the quantitative determination of various mycotoxins of significance for food and feed safety (Figure 2). These sensors employ biological recognition units such as Abs, MIPs, or Apts. We discuss the diversity of detection techniques and technical approaches in these developments, highlighting their wide range of application areas. It is important to note that our presentation, organised by the target analyte mycotoxins, is not exhaustive, but rather aims to provide an overview of these advancements.

## 2. Biosensors Based on Antibodies

Antibody-based biosensors, also termed immunosensors, constitute a distinct category within the realm of biosensors. In these biosensors, the design involves immobilising a biologically sensitive substance, which can be either the antigen or antibody required for the given test. Due to the high selectivity of the immune reaction, manifested in the binding between antigen and antibody, this forms the basis for analytical methods that enable the quantification of these components. Immunoanalytical tests often employ selective antibodies extracted from the blood serum of vertebrate animals, such as rabbits, mice, rats, sheep, horses, goats, and chickens [42].

The antibody molecule is a multifunctional protein characterised by a unique structure that enables specific binding to antigens. However, the immune system elicits a response only to immunogens with a molecular weight exceeding 5 kDa. Smaller molecular compounds (haptens) do not independently trigger antibody production. Yet, when coupled with a large molecular carrier, they can be transformed into complete antigens (immunogens). Typically, large protein molecules serve as carriers, and immunisation, resulting in protein conjugates, is used for the purpose. Nonetheless, the production of sufficiently selective and sensitive antibodies is often time-consuming, expensive, and subject to variation based on the immunisation process. Moreover, issues like poor solubility may arise [43]. To overcome these difficulties, researchers have explored various types of monoclonal or genetically engineered antibodies for biosensors. Both polyclonal and monoclonal antibodies (pAbs and mAbs) react with the same antigen. The primary distinction between them lies in the fact that pAbs are produced by a native set of different plasma B cells and bind to distinct epitopes in the same antigen, whereas mAbs are produced by a single clone of plasma B cells and bind to a unique epitope. The advantage of mAbs over pAbs is that antibodies of given specificity and isotype can be produced with constant quality (Table 1).

Immunosensors can also be categorised based on the approach employed to detect the complex formed during the immune reaction [38]. Since the formation of this complex typically lacks an easily measurable product, some kind of labelling is necessary for determination. This can involve the use of a radioactive isotope, enzyme, fluorescent or chemiluminescent molecule, and, more recently, magnetic NPs. Depending on the measurement method, these labels can be attached to either the antibody (utilised in immobilised antigen-based competitive methods and the so-called sandwich method) or the antigen (employed in immobilised antibody-based competitive methods). Ab-based biosensors for mycotoxins, which are discussed in detail below, are listed in Table 2, categorised by their target analyte mycotoxin.

Riberi et al. [44] reported an EC immunosensor for determining PAT in apple juice. It consists of a glassy carbon electrode (GCE) modified with a dispersion of graphene oxide (GO) and polyclonal anti-PAT antibodies. PAT determination was performed using electrochemical impedance spectroscopy (EIS). The linear calibration range was 0.01–10 ng/mL, and the LOD and the concentration of PAT used to obtain mean (50%) inhibitory concentration (IC_50_) were 9.8 pg/mL and 360 pg/mL, respectively.

Nabok et al. [45] reported a planar waveguide polarisation interferometer immunosensor detecting OTA at 10 pg/mL level. 

Hou et al. [46] designed an OTA EC immunosensor with a new approach. The chemically synthesized mimotope peptide of OTA phage was used as a mimic of the traditional antigen in a competitive sensing platform. The working GCE electrode was modified by the anti-OTA mAb under optimised test conditions (anti-OTA mAb/Mal-polyethylene glycol-NH_2_/MPA/AuNPs/GCE). The developed immunosensor, applying square-wave voltammetric (SWV) detection, had a LOD of 2.04 fg/mL and a linear range of 7.17–548.76 fg/mL. Furthermore, the accuracy of this developed immunosensor was evaluated by testing spiked beer and corn samples, which showed low matrix interference.

Nabok et al. [47] developed a silicon oxide/ silicon nitride/ silicon oxide (SiO_2_–Si_3_N_4_–SiO_2_) waffle-based waveguide polarisation interferometer immunosensor for the detection of AFB1. The sensor setup similar to that of the Mach-Zehnder interferometer allowed detection of AFB1 at 10 pg/mL level.

Srivastava et al. [48] developed a label-free immunosensor for the EC detection of AFB1 using reduced GO (rGO-Ni NPs) sheets decorated with nickel NPs. The rGO-Ni NPs were electrophoretically deposited on a glass electrode coated with indium tin oxide (ITO). The linear measurement range of the prepared immunosensor was 1–8 ng/mL AFB1, while the LOD was 0.16 ng/mL AFB1. The possibility of achieving a low LOD was attributed to the highly crystalline nature of the rGO-Ni NPs sheets, along with the excellent electrocatalytic properties of the Ni NPs. These factors contributed to a better rate of heterogeneous electron transfer. The favourable environment for antibody conjugation on the surface of the rGO-Ni NP sheets also contributed to the LOD value.

Chen et al. [49] reported a new and highly effective nanoprobe based on specific immunology and modified UCNPs developed for the simultaneous detection of mycotoxins. Rare earth-doped UCNPs (NaYF4:Yb/Ho/Gd and NaYF4:Yb/Tm/Gd) have a promising potential in biological detection due to their unique frequency upconversion ability and high detection sensitivity. During the procedure, a competitive immunosensor was developed for the parallel examination of AFB1 and DON by immobilising the corresponding conjugates with magnetically induced separation of UCNPs and the specific formation of an antibody-target complex. Under optimised conditions, the advanced fluorescence probes showed stronger fluorescent properties, broader biological applications, and better storage stability compared to conventional UCNP-based ones. Ultrasensitive determination of AFB1 and DON was achieved in a wide detection range of 0.001–0.1 ng/mL with a LOD of 0.001 ng/mL. In addition, the applicability of the improved nanosensor for the detection of mycotoxins in adulterated peanut oil samples was also confirmed.

For the detection of ZON, a capillary-based immunofluorescence sensor was developed and integrated into a flow injection analysis system [50]. The light-conducting capillary was axially illuminated by a 473 nm/5 mW solid-state laser through an optofluidic connector, and the system showed high sensitivity by efficiently collecting and detecting the undirected fluorescence signal scattered along the capillary wall. The glass capillary used as a measuring cell was silanised with 3-aminopropyltriethoxysilane in the liquid phase in order to fix the biomolecules, and the ZON conjugate to bovine serum albumin (BSA) was immobilised with glutaraldehyde inside the capillary. A competitive fluorescence measurement system was developed for the quantitative determination of ZON, where a 2 µg/mL dilution of the primary antibody stock solution and a 1:2500 dilution of the secondary antibody solution carrying the fluorescent dye were set. The developed capillary-based immunosensor enabled a LOD of 0.003 ng/mL and a LOQ of 0.007 ng/mL for ZON in a competitive immunosensor setup with a dynamic detection range of 0.01–10 ng/mL.

Nabok et al. [51] developed a planar waveguide polarisation interferometer immunosensor for the detection of ZON. Abs were anchored to the sensor surface using immobilized protein A on a deposited polyelectrolyte layer, and the sensor setup allowed detection of ZON at 10 pg/mL level.

Székács et al. [52] successfully developed a label-free competitive immunosensor for the determination of ZON based on the OWLS measuring technique. Depending on the covalent immobilisation method applied, an outstanding LOD of 0.002 pg/mL was obtained for ZON in the competitive immunosensor setup with a dynamic detection range between 0.01 and 1 pg/mL. ZON concentrations with high selectivity were proven using structural analogues of ZON. The method was applicable for monitoring ZON in maize.

Adányi et al. [53] developed a competitive OWLS-based immunosensor for the determination of AFB1 and OTA mycotoxins from wheat and barley samples. The AFB1-conjugate was immobilised by glutarealdehyde on the sensor chip functionalised by amino groups, and the proper diluted mAb was mixed with the sample before injection. Using the optimised procedure for AFB1 and OTA analysis, dynamic measuring ranges of 0.001–1 ng/mL and 0.5–10 ng/mL were obtained and the IC_50_s were 0.023 ± 0.009 ng/mL and 2.01 ± 0.47 ng/mL, while the lower LOD was found to be 0.0005 ng/mL and 0.1 ng/mL, respectively. When testing the wheat, barley, and paprika samples, the results measured with the immunosensor corresponded to the values measured with the enzyme-linked immunosorbent assay (ELISA) reference method at the *p* < 0.05 significance level based on an independent two-sample t-test.

The OWLS technique has been applied to label-free detection of AFB1 in a competitive immunosensor for the detection of AF in the spice paprika matrix [54]. The measuring range of AFB1 was found to be 0.1–100 ng/mL when measuring spiked paprika samples. The AFB1 content of sixty commercial spice paprika samples from different countries was measured with the developed and optimised OWLS immunosensor. Comparing the results from the indirect immunosensor with those obtained using HPLC or ELISA showed excellent correlation.

To compare the analytical sensitivity achieved with an immunosensor design allowing signal enhancement by increasing the sensor surface through immobilisation of gold nanoparticles (AuNPs) of different size and origin obtained via chemical or biotechnological synthesis, OWLS was applied for the detection of AFB1, in a competitive ELISA format [55]. The effect of AuNPs median size, the methods of sensitisation, and the biochemical parameters of the immunosensor were examined. The novel immobilisation technique applied for the detection of AFB1, using a competitive immunoassay, had a dynamic range of 0.01–10 ng/mL with an IC_50_ of 0.044 ± 0.005 ng/mL.

Majer-Baranyi et al. [56] successfully developed a selective immunosensor for determining the DON content of wheat. A conjugate was prepared from the DON mycotoxin after sodium periodate treatment using ovalbumin (OVA) and BSA. A pAb was prepared using the DON–OVA conjugate. The competitive OWLS-based immunosensor was developed, and the dynamic measurement range was found to be 0.005–50 ng/mL and the IC_50_ was 0.15 ± 0.08 ng/mL, while the lower LOD was 0.001 ng/mL. Based on the calibration curve obtained with wheat flour samples spiked with DON, the dynamic measurement range calculated for the flour sample was 0.01–10 mg/kg and the IC_50_ was 0.13 ± 0.04 mg/kg, which met the detection requirements set in the corresponding regulations.

Since mycotoxins are typically not found alone but as multiple contaminants in different crops, foods, and feeds, the immunosensors suitable for multiplex mycotoxin determination should be highlighted separately, as their use is suitable to significantly speed up the tests. Lu and Gunasekaran [57] developed an EC immunosensor applicable for simultaneous monitoring of multiple mycotoxins, such as FB1 and DON. These two mycotoxins are widely present as co-contaminants in raw food materials. A two-channel and three-electrode EC sensor was fabricated using photolithography etched on transparent ITO-coated glass and attached to a capillary-driven polydimethylsiloxane (PDMS) microfluidic channel. The two working electrodes were functionalised with AuNPs and anti-FB1 and anti-DON Abs. With this dual-channel ITO microfluidic EC immunosensor, the corresponding linear detection range was 0.3–140 pg/mL and 0.2–60 pg/mL, while the LOD was 97 pg/mL and 35 pg/mL for FB1 and DON, respectively. The EC sensor remained stable for two weeks under proper storage conditions, and its performance was tested with ground corn extract as a real food matrix.

Wei et al. [58] developed an SPR method for fast and high-throughput detection of mycotoxins in corn and wheat samples. The antigen was immobilised on the sensor as a self-assembled monolayer (SAM) in the form of a hydrazone bond. The LODs for AFB1, OTA, ZON, and DON were 0.59 ng/mL, 1.27 ng/mL, 7.07 ng/mL, and 3.26 ng/mL, respectively.

Joshi et al. [59] presented a portable nanostructured imaging SPR (iSPR) device, with a 6-plex competitive inhibition immunoassay to investigate mycotoxin contamination of barley. In order to create the biologically sensitive surface of the sensor suitable for measuring DON, ZON, T-2 toxin, OTA, and FB1, OVA conjugates of mycotoxins were immobilised on the chip via amine coupling. The SPR response was then obtained via injecting a mixture of fixed concentrations of antibodies and the sample (of matrix-matched standard) onto a chip containing immobilised mycotoxin–OVA conjugates. The sensors could be used for at least 60 cycles with regeneration after the samples. The LODs in barley were 26 μg/kg for DON, 6 μg/kg for ZON, 0.6 μg/kg for T-2, 3 μg/kg for OTA, 2 μg/kg for FB1, and 0.6 μg/kg for AFB1. The preliminary internal validation showed that DON, T-2, ZON, and FB1 could also be detected at the European Union regulatory limit values, while in the case of OTA and AFB1, the sensitivity needed to be improved. Furthermore, measurement of naturally contaminated barley showed that the assay could be used as a semi-quantitative screening method for mycotoxins.

Hossein et al. [8] developed and validated a fast, sensitive, and multiplex iSPR biosensor assay for the detection of three *Fusarium* toxins, DON, ZON, and T-2. The iSPR assay was based on a competitive inhibition format with AuNP-conjugated secondary antibodies used as amplification labels. The antigen-coated sensor chip was used for more than 46 cycles without a significant decrease in signal intensity (<12%). Matrix-fitted calibration curves were used to determine *Fusarium* toxins in wheat. The LOD was 15 µg/kg for DON, 24 µg/kg for ZON, and 12 µg/kg for T-2 toxin. Average recoveries ranged from 87% to 103%, and the relative standard deviation of repeatability was less than 5%. The LOD values of all three *Fusarium* toxins were validated with spiked wheat contaminated at the maximum level regulated by the EC (100 µg/kg for ZON and T-2 toxin, while 400 µg/kg for DON), and it was found that the developed sensor provided sufficient sensitivity to monitor the contamination of wheat with these mycotoxins.

A TRFM immunochromatographic test strip (TRFM–ICTS) combined with ultraviolet light was developed for the rapid and quantitative detection of AFM1 in milk and its products [60]. In order to generate the signal, time-resolved fluorescent europium (III) [Eu(III)-TRFM] polystyrene microspheres as markers were conjugated to a mAb. Following preincubation for competitive recognition, under optimal conditions, the TRFM–ICTS had a linear range of 0.05–2.0 ng/mL and an IC_50_ of 0.204 ng/mL, while a LOD of 0.019 ng/mL occurred. The TRFM–ICTS showed good recovery values ranging from 84.6% to 119.0% in spiked samples containing milk powder and pasteurised or ultra-high-temperature processed milk.

A time-resolved fluorescence immunochromatographic assay (TRFICA) was developed with idiotype nanobodies for simultaneous detection of AFB1 and ZON in corn commodity and products [61]. The Eu/Tb(III) nanosphere with enhanced fluorescence was conjugated to the anti-idiotypic nanobody (AIdNb) and mAb was used as a label. Based on the nanosphere-antibody conjugation, two competitive time-resolved strip methods (AIdNb–TRFICA and mAb–TRFICA) were developed and compared. The IC_50_ using AIdNb–TRFICA was 0.46 and 0.86 ng/mL for AFB1 and ZON, which were 18.3 and 20.3 times more sensitive than the mAb–TRFICA methods, respectively. The AIdNb–TRFICA was also applied for dual detection of the mycotoxins and provided quantitative correlations of 0.13–4.54 ng/mL for AFB1 and 0.20–2.77 ng/mL for ZON, with LODs of 0.05 and 0.07 ng/mL, respectively.

Dorokhin et al. [62] described an iSPR-based multiplex microimmunoassay for the investigation of several mycotoxins. A competitive mycotoxin–protein conjugate microarray-based inhibition immunoassay system in a flow microspotter format was developed for the simultaneous detection of DON and ZON using a single sensor chip. The validation showed LODs of 21 and 17 ng/mL for DON and 16 and 10 ng/mL for ZON in the extracts, corresponding to 84 and 68 μg/kg for DON and 64 and 40 μg/kg for ZON in corn and wheat samples, thus meeting the regulatory limits of the European Union.

Although the paper-based lateral flow immunoassay (LFIA) is widely used in mycotoxin monitoring, ultrasensitive and quantitative detection is still an enormous challenge. Cai et al. [63] examined single-atom nanozymes (SAzymes) and the optimal Fe-N-C SAzyme with highly efficient catalytic performance was successfully used as a marker and catalytic signal amplifier in LFIAs based on mAb for the detection of FB1 and AFB1 mycotoxins. Qualitative and quantitative detection could be easily performed with the catalytic amplified system. By observing the test lines with the naked eye or a smartphone, the linear measurement range was found to be 0.02–150 ng/mL for FB1 and 0.005–200 ng/mL for AFB1, while the LOD was 0.0139 and 0.0028 ng/mL, respectively.

Zhang et al. [64] developed a multiplex SERS-based lateral flow immunosensor for six major mycotoxins in maize. Two characteristic Raman reporter molecules (5,5′-dithiobis-(2-nitrobenzoic acid) (Ellman reagent) and 4-mercaptobenzoic acid) were used to label the synthesized Au@Ag core-shell nanoparticles to prepare SERS nanoprobes as detection reagents. Six corresponding hapten–protein conjugates were used on three test lines on nitrocellulose membrane, with two conjugates as capture antigens on each line. This design facilitated the simultaneous detection of six mycotoxins in a single test. After optimising the experimental parameters of the immunosensor, the LODs were found to be 0.96 pg/mL for AFB1, 6.2 pg/mL for ZON, 0.26 ng/mL for FB1, 0.11 ng/mL for DON, 157 pg/mL for OTA, and 8.6 pg/mL for T-2 toxin. The spike experiment showed high accuracy with a recovery of 78.9–106.2%. The test could be performed in less than 20 min, and the measurement results corresponded to those measured with the HPLC-MS technique.

Instead of the previously used colloidal gold and coloured latex labels, GO and carboxylated GO were used as labels for LFIAs, aiming to improve sensitivity [65]. The developed sensor enabled detection and rapid screening of AFB1, where the antibody-GO complex was used as a recognition element. The visual LOD and threshold for AFB1 were 0.3 ng/mL and 1 ng/mL, respectively. The quality test took 15 min, and the analytical results showed good agreement with the reference LC MS/MS method. The method was successfully used for on-site determination of AFB1 in peanut oil, maize, and rice samples.

A rapid LFIA test strip with fluorescent microspheres (FM) was investigated for the detection of DON residues in various agricultural products. For the competitive immunoreaction, ultrasensitive anti-DON mAbs were produced and covalently linked to carboxylate-modified FMs, which were used as markers in a competitive immunochromatographic assay. Under optimal conditions, the visual LOD of DON using FM LFIA was 2.5 ng/mL and the threshold was 25 ng/mL, while the recovery of spiked DON in agricultural samples was 90.20–107.32% [66].

Cai et al. [22] developed an anti-TeA McAb-based test strip for rapid testing of TeA. For the optical probe, gold nanoparticles (AuNPs; average diameter 17.25 nm) and, to further improve the sensitivity, multibranched gold nanoflowers (AuNFs; average diameter 50 nm) were prepared and used to label anti-TeA McAb. The AuNP-based strip had an assay time of 15 min, a visual LOD of 12.5 ng/mL, and a threshold of 100 ng/mL, while the AuNF-based strip had a visual LOD of 0.78 ng/mL and a threshold of 50 ng/mL. Both test strips were used to determine TeA in apple juice and tomato ketchup, and the results were consistent with results obtained using the UHPLC-MS/MS method.

## 3. Biosensors Based on Molecularly Imprinted Polymers (MIPs)

MIPs are biomimetic synthetic receptors that have specific recognition cavities complementary in shape, size, and spatial arrangement to given template molecules, similar to a “key and lock” mechanism. These cavities are formed in a highly cross-linked polymer matrix. MIPs are synthesized through the copolymerisation of functional monomers and cross-linkers in the presence of target molecules, which act as templates. These templates are subsequently removed after the polymerisation process is completed. The resulting imprinted cavities or recognition sites behave as non-biological receptors [67,68].

MIPs can be employed as recognition elements in MIOMs either in the form of MIP-based particles, which can be synthesized using various methods such as bulk polymerisation, precipitation polymerisation, emulsion polymerisation, solid-phase polymerisation, sol-gel polymerisation, and surface imprinting, or in the form of MIP-based films on transducer surfaces or in piezoelectric systems created via electropolymerisation, photo-induced polymerisation, or enzyme-induced polymerisation [69]. The thickness of MIP films can be controlled by adjusting the polymerisation conditions. MIPs offer several advantages, including high specificity and sensitivity, ease of operation, cost-effectiveness, long shelf-life, and inherent stability. Their high stability in harsh physical and chemical environments, along with their inherent molecular recognition abilities, make MIPs a promising alternative to bioreceptors. MIPs have found applications primarily in EC tests, QCMs, and optical biosensors [70] for the detection of various mycotoxins. One of the most important areas for the application of MIPs is food analysis [71].

For the determination of mycotoxins, optical transducers, including fluorescent sensors, are the most common among MIP-based sensors. They can detect intrinsically fluorescent target molecules, but, in the case of non-fluorescent analytes, the application of a fluorescent component is required. In both cases, there are some drawbacks such as interfering with analytes with similar fluorescent emission or tedious procedures for introducing fluorescent components into MIP. To overcome these shortcomings, MIP sensors can be functionalised with different kinds of QDs (CdTe-, CdSE-, ZnSe-, ZnS-, Mn- and Cu-doped ZnS-, and carbon QDs) [72,73,74], UCNPs, or with MOFs [75]. The combination of the remarkable sensitivity of the NPs with the selectivity of MIPs allows the formation of MIP composites of high sensitivity and selectivity. MIP-based biosensors for mycotoxins, which are discussed in detail below, are listed in Table 3, categorised by their target analyte mycotoxin.

MIP-coated CdTe QDs were prepared for a rapid determination method of AFB1 by Guo et al. [76]. MIPs were prepared using sol-gel polymerisation with dummy template technology. The fluorescent sensor showed a linear measuring range for AFB1 between 80 and 400 ng/g. With the optimised sensor, AFB1 content in spiked coix seed and wheat seed was determined. The recoveries ranged from 99.2% to 101.8%.

Chmangui et al. [77] used Mn-doped ZnS QDs modified with polyethylene glycol anchored on the surface of a MIP layer synthetized using dummy template technology to create a fluorescent probe for AF determination in almond-, soy-, and rice-based beverages. The sensor has been successfully applied as a screening method for the assessment of total AF content in non-dairy milk samples. The sensor offered a LOD of 16 ng/mL, close to the maximum permissible level of total AF content set in the legislation of the European Union.

A carbon dots (CD)-embedded MIP sensor using fluorescent detection was developed for STC determination by Xu et al. [78]. In their experiment, luminescent carbon dots were coated with MIP via a non-hydrolytic sol-gel process, where 1,8-dihydroxyanthraquinone was used as the template molecule to detect STC. The sensor exhibited high sensitivity and selectivity. The LOD of STC was 0.19 μg/mL, and the linear measuring range was between 0.05 and 2 μg/mL. It was used to determine the STC content of different grain samples such as millet, maize, and rice.

Shi et al. [79] demonstrated the usefulness of β-cyclodextrin-functionalised carbon nanosheets modified with MIPs, using the structural analogue 1,8-dihydroxyanthraquinone as a substitute template, for sensitive and selective fluorescence detection of STC. The fluorescent signal decreased linearly in the 48.6 ng/mL to 1 μg/mL STC concentration range, and the LOD was 24 ng/mL.

A fluorescence sensor, where carbon QD-encapsulated molecularly imprinted fluorescence-quenching particles were used, was presented by Shao et al. [80]. With the established sensor, ZON content of maize samples purchased from the local market was determined. During the measurement, the fluorescence of carbon dots is quenched if ZON is present in the sample; therefore, the fluorescence signal is inversely proportional with the concentration of ZON. The LOD with the optimised sensor was 20 ng/mL and the linear working range was between 0.02 and 1.0 μg/mL.

A novel MIOM based on ionic liquid-stabilised CdSe/ZnS QDs was developed for the highly selective and sensitive detection of ZON [81]. The ZON analogue cyclododecanyl-2,4-dihydroxybenzoate was used as a template. Under optimised operating parameters, the linear detection range of ZON was 0.955–993 ng/mL and the LOD was 0.64 ng/mL, applicable in corn, rice, and wheat flour. ZON recovery ranged from 84.0 to 106.0% at three concentrations, 50, 100, and 500 ng/g.

Researchers have developed an optosensor for the extremely sensitive detection of ZON in wheat by coating a MIP layer on the surface of a luminescent MOF with cyclododecanyl-2,4-dihydroxybenzoate, using the analogue of ZON as a template, and 3-aminopropyltriethoxysilane as a functional monomer [82]. Under optimised operating conditions, the relative fluorescence intensity of the magnetic MIP decreased linearly, the linear measurement range of ZON was 0.05–1.0 µg/mL, and the LOD was 0.018 µg/mL, while the recovery was 95.5–98.0% (RSD < 4.7%). The stable and selective sensor successfully detected ZON in wheat.

Bagheri et al. [83] presented a fluorescent PAT sensor where a MIP layer was formed on the surface of a AgNPs/Zn-based MOF composite, combining the outstanding peroxidase-like catalytic activity of the MOF composite with the high selectivity of the MIP for PAT. During the measurement, the MIP-capped Zn-MOF composite catalysed the hydrogen peroxide–terephthalic acid reaction, resulting in a highly fluorescent product; therefore, in the presence of PAT, the fluorescence emission was quenched proportionally to the PAT concentration. Fluorescence intensity decreased linearly within the range of 0.015–1.541 ng/mL and the LOD was 9.2 ng/mL for PAT.

Combining the high selectivity of MIP with the high fluorescence intensity of UCNPs, Yan and Fang proposed a fluorescent MIP sensor for OTA determination [84]. The MIP was developed on silica-coated UCNPs using N-(1-hydroxy-2-naphthoylamido)-(L)-phenylalanine as an alternative template. In the presence of OTA, the fluorescence of the UNCPs-MIP was quenched; therefore, the intensity of the fluorescence signal was inversely proportional to the concentration of OTA. With the optimised sensor, a linear working range of 0.05 and 1 μg/mL, with a LOD of 31 ng/mL could be achieved for OTA.

Sergeyeva et al. [85] fabricated an AFB1-selective MIP membrane via UV-initiated radical polymerisation using ethyl-2-oxocyclopentanecarboxylate as a dummy template. The MIP-based fluorescence sensor provided highly selective detection of AFB1 within the range of 14–500 ng/mL with a LOD of 14 ng/mL. The sensor was applied for the analysis of waste waters from a bread factory. The smartphone-based MIP-based fluorescence sensor was prepared for point-of-care detection of AFB1 via UV-induced polymerisation using a dummy template. The fluorescent signal of AFB1 adsorbed on the surface of the MIP was detected using a smartphone camera after excitation with UV irradiation. The intensity of the fluorescence signal was directly proportional to the AFB1 content. The dynamic measuring range of the sensor system for AFB1 comprised 20–100 ng/mL. The storage stability of the MIP film at 22 °C was estimated to be as low as one year [86].

A disposable evanescent wave fibre-optic sensor was developed for the determination of CIT, where a 4 cm long polystyrene optical waveguide was coated with a MIP containing a fluorescent signalling group [87]. The fluorescent signal is provided by the MIP containing the naphthalimide-based fluorescent monomer upon binding to the molecules containing the carboxyl group. MIP coating was carried out ex situ, via immersing the fibre with pre-synthesized MIP particles, or in situ, using photopolymerisation on the fibre. An increase in fluorescence was observed at CIT concentrations of 0.5–2.5 μg/mL.

A luminescent sensor with porous MIP microspheres doped with Eu(III) was prepared for selective fast analysis of TeA, produced by *Alternaria* species, in rice extracts. The resulting Eu(III)-imprinted polymer had a strong, long-lived red emission when excited in the near-UV (337 nm) in the presence of the mycotoxin. The luminescence intensity was monitored at 615 nm. With the optimised senor, a linear response between 1.7 and 20 μg/mL was obtained, with a LOD of 0.5 μg/mL [24].

In contrast, Quílez-Alburquerque et al. [88] used Ru(II)-doped MIP nanocomposites to detect TeA. The luminescent MIP nanoshell was grown onto a 200 nm silica core allowing fast diffusion of the analyte through the MIP shell; as a result, the real-time detection of the mycotoxin became possible. The probe luminescence lifetime as a function of the analyte concentration was monitored. With this novel sensor set-up, a more sensitive detection has been achieved for TeA determination in the concentration range of 0.098–78.89 μg/mL, with a LOD of 63.8 and 75.2 ng/mL for steady-state and time-resolved luminescence measurements, respectively.

The SPR technique has recently received remarkable attention as a label-free optical detection technique used for real-time monitoring and analysis of biomolecular interactions. SPR is used to determine the target analytes of complex matrices by immobilising biospecific components and often allows, with minimal sample preparation requirements, high specificity and selectivity. It provided information on the affinity, specificity, and kinetic parameters of biomolecular interactions. Furthermore, the imaging capability of iSPR allows users to visualize the entire workspace and work in a multiplexed format.

Akgönüllü et al. [89] reported a label-free and selective SPR-based sensor for the detection of OTA contamination in dried figs. A MIP film was deposited on the SPR sensor chip via light-induction polymerisation of N-methacryloyl-(L)-phenylalanine and 2-hydroxyethyl methacrylate in the presence of OTA as a template. The developed MIP sensor exhibited a wide linear measurement range of 0.1–20 ng/mL, while the LOD was 0.028 ng/mL.

A MIP-SPR nanosensor has been presented for AFB1 determination in corn samples. A gold sensor surface was modified with MIPs embedded on AuNPs to enhance sensitivity and selectivity towards the low-molecular-weight analyte. The imprinted nanosensor showed a wide linear range between 0.1 pg/mL and 10.0 ng/mL, with a LOD of 1.04 pg/mL. The sensor was applied for the examination of corn and peanut samples [90]. Based on the above-mentioned application, the research group developed another sensor where AuNPs decorated with a MIP nanofilm were immobilised on the gold sensor surface for AFM1 determination. The sensor was utilised for determining AFM1 content of raw milk samples. The dynamic measuring range was between 0.3 pg/mL and 20 ng/mL, and a LOD was 0.4 pg/mL for AFM1 [91].

Choi et al. [92] developed a sensor for the detection of DON based on an SPR transducer using the MIP technique. A template was formed in the presence of DON as a template, and the MIP film was produced on a bare Au chip via the electropolymerisation of pyrrole. The sensor was suitable for detecting DON in the linear range of 0.1–100 ng/mL, while its LOD was >1 ng/mL.

For CIT determination in red yeast rice, a CIT-imprinted SPR sensor was developed by Atar et al. [93]. On the allyl mercaptan-modified gold sensor surface, a CIT-imprinted film was generated. The biosensor showed high sensitivity towards CIT with a linear working range of 0.005–1.0 ng/mL and a LOD of 1.7 pg/mL. The sensor was used to determine the CIT content in red yeast rice.

Wu et al. [94] developed a specific and sensitive PAT sensor based on the MIP and SERS techniques. AuNPs were synthesized for the MIP-SERS sensor as a carrier, with 4-vinylpyridine as a functional monomer, 1,4-diacryloylpiperazine as a cross-linker, PAT as a template molecule, and horseradish peroxidase (HRP) as an initiator. Due to the good SERS character of AuNPs and the excellent selectivity of MIP, the MIP-SERS sensor linearity operating range (R2  =  0.988) was between 0.001 and 7.7 ng/mL PAT, while the LOD was 0.828 ng/mL (S/N  =  3). The method was applied during fruit product control and the recovery was 96–108%.

QCM-based sensors monitor the shift in the resonance frequency of the quartz crystal in the microbalance device due to mass increase caused by the absorption of analytes onto the sensor. It enables label-free and real-time detection and determination of mass changes at nanogram-level on the cantilever surface of the QCM, both in gas and liquid phases.

For PAT determination, a QCM-based sensor was developed using four structural analogues of PAT to facilitate the reduction of mycotoxin levels in food production processes, while also dropping the cost of manufacturing. The MIP was prepared through a sol-gel MIP copolymerisation process. With the established sensor, the PAT content of spiked food samples was determined. The linear measuring range of PAT detection was 7.5–60 ng/mL with a low LOD of 3.1 ng/mL. The utility of the sensor was verified by measuring PAT levels in apple and pear juice and haw flakes [95].

Gu et al. [96] developed a QCM-MIP sensor for AFB1 determination using AuNP-doped MIP- and AuNP-modified covalent organic framework composites in order to provide large surface area; therefore, more recognition sites could be created to enhance sensitivity. The sensor showed a wide linear range (0.05–75 ng/mL), with a LOD of 2.8 pg/mL. With the optimised sensor, AFB1 content of spiked peanut, pistachio, rice, and wheat samples were successfully determined.

A QCM-based MIP sensor for CIT determination in cereal samples was presented using a poly(2-aminothiophenol)-MIP membrane as the recognition element on the Au electrode surface modified with a functional composite of AuNPs and mesoporous carbon by Fang et al. [97] In a range between 1.5 and 50.1 ng/mL, the sensor showed a frequency shift linearly correlated with the concentration of CIT, with a low LOD of 0.45 ng/mL.

The advantages of MIP-based EC sensors include high selectivity and sensitivity, chemical stability, low cost, and miniaturisation, making these devices a topic of significant interest in the field of food analysis [53]. The introduction of functionalized nanomaterials/NPs, thanks to their significantly increased surface area and to the improved conductivity of various electrodes, plays a crucial role in the development of EC sensors. Combining conductive polymers with metal NPs creates a metal NP-embedded nanomatrix structure that enhances electron transfer, expands the electroactive surface area of the electrode, and increases signal strength and long-term stability [77]. Conductive polymers are employed to modify sensor characteristics such as speed, sensitivity, and efficiency in detecting analytes. They are promising materials due to their excellent EC conductivity, low optical transition, low ionization potential, and high electron affinity. The combined use of conductive polymers and NPs has emerged as a leading direction in the development of EC MIP sensors, thanks to their beneficial effects.

Hatamluyi et al. [98] introduced an ultrasensitive and selective EC MIP sensor for PAT determination. The MIP using PAT as a template was synthesized via electropolymerisation on the surface of the GCE modified with nitrogen-doped graphene QDs and a AuNP-functionalised Cu-MOF. With the optimised sensor, the PAT concentration of apple juices was investigated using differential pulse voltammetry (DPV) by recording the electrode response in the potential range between −0.4 to +0.2 V at a scan rate of 50 mV/s. The sensor presented high sensitivity with a linear range of 0.001–70.0 ng/mL, a low LOD of 0.0007 ng/mL, and good selectivity for PAT over AOH, OTA, and AFB1.

In another experiment, a Co-based MOF loaded with selenium disulfide, as well as a Au nanocomposite on polyalanine, with a MIP using PAT as template was synthetised and immobilised on the surface of the carbon-based screen-printed electrode to create an EC sensing platform for PAT determination. With the established sensor, a LOD of 0.102 pg/mL and a wide dynamic measuring range (0.154 pg/mL–15.4 ng/mL) for PAT was achieved. The sensor possessed excellent reusability and stability [99].

Guo et al. [100] fabricated a MIP sensor using 4-aminothiophenol as the functional monomer in the presence of 2-oxindole as the dummy template deposited on the surface of a GCE modified with CDs, chitosan, and AuNPs for the determination of PAT in fruit juices. The linear response range of the DPV measurement with the MIP sensor for PAT determination was from 0.154 pg/mL to 0.154 ng/mL with an extremely low LOD of 0.117 pg/mL.

An innovative method, based on a surface-functionalised monomer-directing strategy, was developed to construct a sensitive and selective molecularly imprinted EC sensor for PAT recognition. A poly(thionine) film coated with PAT-imprinted platinum nanoparticles (PtNPs) with high capacity and fast kinetics was synthesized on the preformed thionine tail surface of PtNP-nitrogen-doped graphene via electropolymerisation. In this process, thionine, which possesses two amino groups, acted as both a functional monomer in the MIP and also a signal indicator. The functional monomer demonstrated a good electron transfer ability. The combination of PAT and MIP led to a reduction in the electrical conductivity of the MIP film, resulting in a decreased reduction peak current. Consequently, the sensor exhibited excellent performance for the detection of PAT within the range of 0.002–2 ng/mL, with a LOD of 1 pg/mL. The feasibility of this approach was demonstrated by successfully detecting PAT in apple and grape juice samples [101].

A MIP-based EC sensor was developed for the determination of AFB1 and FB1. Polyaniline (PANI) was employed as a monomer, and mycotoxin molecules served as a template for MIP synthesis using the chemical oxidative polymerisation method. MIP films were deposited onto an ITO-coated glass substrate using the electrophoretic deposition technique to create MIP electrodes. DPV was utilised for EC sensing of AFB1 and FB1. The fabricated sensor exhibited LODs of 0.313 and 0.322 pg/mL for AFB1 and FB1, respectively. The sensor demonstrated good linearity within the range of 1 pg/mL to 500 ng/mL when testing spiked corn samples [102].

Another EC sensor platform was fabricated for the determination of FB1. In this sensor set-up, a nano-MIP (used as a recognition element) was immobilised on the surface of the Pt electrode modified with polypyrrole-zinc porphyrin composite. The FB1 content of maize samples was determined by applying DPV. The linear measuring range was between 0.72 fg/mL and 7.22 pg/mL of FB1 with an extremely low LOD of 0.02 fg/mL [18].

Radi et al. [103] used a molecularly imprinted poly(o-phenylenediamine) (PPD) film deposited on a screen-printed gold electrode surface to create an impedimetric sensor for the determination of ZON in corn flake samples. The sensor showed a wide determination range from 2.5 to 200 ng/mL for ZON, with a LOD of 0.20 ng/mL.

A sensitive EC sensor with molecular imprinting was prepared for the selective detection of T-2 mycotoxin. To increase the selectivity and sensitivity of MIPs, chelation in MIPs was improved with iron ions (Fe^3+^), and an EC-printed sensor was developed for the determination of T-2 in cereals and human serum samples using modified Fe^3+^-MIP-GCE for DPV detection. Under optimised analytical conditions, the linear measurement range covers the concentration range from 1.12 nM to 2.12 μM, with a LOD of 0.33 nM (~0.15 ng/g) [16].

Elfadil et al. [21] described a rapid electrochemical determination method for the quantitative analysis of CIT. The method is based on the use of graphene nanoflakes (GFs) prepared via rapid, solvent-free, aqueous phase exfoliation of graphite with sodium cholate. Nanoscale GFs dispersed in water were used as sensing layers of SPEs. The MIP prepared via rapid synthesis (5 min) was used for the selective extraction and purification of CIT from various samples such as red rice, cranberry, turmeric, corn, wheat germ, and rice starch. With the method, the determination of CIT was achieved with a LOD of 5 ng/mL, while the measurement range was 25–12,500 ng/mL with exact recovery (85.8–111.4%) for all samples.

A new MIP-based voltammetric sensor was developed on GCE modified with PtNP and polyoxometalate-functionalised, reduced graphene oxide (rGO) for the determination of CIT. The linearity range of the developed method was 0.25–25 fg/mL, and the LOD was 0.05 fg/mL. The voltammetric sensor was successfully used for detecting CIT contamination in rye samples [104].

To determine CIT, a MIP electrochemical sensor based on PdNP-modified GCE with functionalised graphene quantum dots (GQDs) was developed for CIT analysis. In the presence of 80.0 mM pyrrole as a monomer and 20.0 mM CIT as a template, a CIT-imprinted electrochemical surface was formed on the surface of the sensor. The linearity range of the developed nanosensor was within the range of 250–1250 fg/mL, and the LOD was 50 fg/mL [105].

## 4. Aptamer-Based Biosensors

Biosensors utilising aptamers (Apts) as biorecognition elements, often referred to as aptasensors, were first described in 1996. They have been employed to detect various small-molecule pollutants, including toxins and mycotoxins. Apts are single-stranded DNA or RNA oligonucleotides containing 10–50 variable bases that can fold into distinct three-dimensional conformations, and bind with high affinity to the target analyte, thus creating a highly selective sensor platform. Large libraries of oligonucleotides are available for the synthesis of Apts, allowing for the selection of specific molecular sequences. The development of the SELEX (Systematic Evolution of Ligands by Exponential Enrichment) procedure made it possible to utilise Apts. SELEX is an iterative in vitro selection and amplification method designed for screening large oligonucleotide libraries [106]. During SELEX, specific target molecules are incubated with oligonucleotide libraries, and subsequent separation of binding from non-binding molecules leads to the amplification of selected oligonucleotides. This process results in a new mixture containing nucleic acid molecules with a higher affinity for the target. Apts exhibit binding affinity and specificity similar to mAbs, but they offer numerous advantages over Abs. These advantages include longer shelf life, shorter preparation time at lower cost, greater stability, and the ability to undergo easy modification with dyes and labels without losing affinity. These characteristics make Apts a viable alternative to natural bioreceptors. However, it is worth noting that testing small molecules with Apts can sometimes lead to time-consuming, non-specific interactions, which can limit their widespread use. Despite this, Apt-based biosensors have been widely developed for the detection of various mycotoxins using fluorescent, colourimetric, and EC sensors [107,108].

Among optical aptasensors, the fluorescent detection method is the most widely used due to its high sensitivity, simplicity, ease of detection, and suitability for developing high-throughput tests. Since mycotoxins are small molecules, signal amplification has always been a challenge in sensor development. The use of various nanomaterials such as nanoisland, nanohorns, nanophotonics, and also new enzyme amplification strategies, has become a direction in sensor development. Aptamer-based biosensors for mycotoxins, which are discussed in detail below, are listed in Table 4, categorised by their target analyte mycotoxin.

Nabok et al. [109] reported a total internal reflection ellipsometry-based aptansensor for the detection of OTA. The method allowed an LOD of 10 pg/mL for the analyte, while the association (K_A_) and affinity (K_D_) constants were found to be 5.63 × 10^7^ mol and 1.77 × 10^−8^ mol, respectively.

Liu et al. [110] presented a label-free fluorescent aptasensor for the determination of OTA in the Mongolian milkvetch (*Astragalus membranaceus*). In their design, a photo-induced electron transfer-based “turn off” process was used to quench the fluorescence intensity of CdTe QDs using (N-methyl-4-pyridy)porphyrin. In the presence of OTA, due to a conformational change, the interaction between CdTe QDs and porphyrin derivative is greatly weakened, resulting in the recovery of CdTe QD fluorescence, which was used as an analytical signal to evaluate the concentration of OTA. The linear dynamic range was 0.2–20 ng/mL, and the LOD was 0.16 ng/mL. In another experiment to create a “turn on/off” fluorescent sensor for OTA determination in coffee and milk, they used ZnCdSe QDs and self-assembled zinc porphyrin, where the porphyrin derivative quenched the fluorescence of ZnCdSe QDs, and in the presence of OTA, the fluorescence of the QDs was recovered. The linear working range of the sensor was between 0.5 and 80 ng/mL, with a LOD of 0.33 ng/mL [111].

Guo et al. [112] also demonstrated a label-free aptasensor for OTA determination using SYBR gold fluorescent dye to enhance fluorescent intensities using single-walled carbon nanohorns (SWCNHs) as fluorescence quenchers. In the presence of OTA, due to a conformational change of the OTA-specific Apt, its adsorption onto carbon NPs was obstructed, leading to recovery of the fluorescence of the dye. The newly developed fluorescent sensor showed a linear response within the concentration range of 5–500 ng/mL, with a LOD of 2.3 ng/mL for OTA.

Hitabatuma et al. [113] developed an aptasensors based on free-complementary DNA for OTA determination in a competitive assay method. In their design, a “molecular beacon” (MB) optical DNA sensor was used, which was functionalised with a fluorophore at one end, and with an amine-reactive quencher, dabcyl, at the other end. In the absence of OTA, the Apt binds to cDNA so the fluorescence of MB is quenched, because the fluorophore and the quencher are in close proximity. But, in the presence of the target analyte, the structure of the MB unfolds so the fluorophore moves further from the quencher resulting in a fluorescence signal. Under the optimised conditions, the sensor exhibited a wide linear working range of 10 pg/mL–1 µg/mL with a low LOD of 0.247 pg/mL.

A highly sensitive signal-amplified fluorescent aptasensor for OTA determination was designed in corn samples using an enzyme-free amplification method by Wang et al. [114]. In their work, a fluorescent perylene probe with dendritic DNA concatemers on amino-modified magnetic NPs (AMNPs) functionalised with OTA Apt was used for OTA determination. In the presence of OTA, due to its high affinity for Apt, the fluorescent probe/DNA composite is released from the magnetic NP producing a strong fluorescent signal. The LOD of the proposed aptasensor was 0.04 pg/mL.

Wu et al. [115] proposed a new FRET-based biological assay to determine PAT. In this research, the sensitivity of rare-earth-doped UCNPs, the selectivity of the Apt, and the attractive advantages of the exonuclease-catalysed target recycling strategy were combined to develop a new biosensor for the determination of PAT in food samples. The linear measurement range of the developed sensor ranged from 0.01 ng/mL to 100 ng/mL, while the LOD and LOQ were 0.003 ng/mL and 0.01 ng/mL, respectively. Furthermore, the average recoveries varied between 93.3% and 105.2% when testing apple juice samples, which confirmed the reliability of this method.

Jo et al. [116] reported a CRET aptasensor for the detection of OTA in roasted coffee beans. The Apt sequences used in this study were 5’-deoxyribozyme-linker-OTA Apt-3’-dabcyl, where dabcyl at the end of the Apt was included as a quencher in the CRET aptasensor. Upon administration of hemin and OTA, the dabcyl-labelled OTA Apt exerts HRP-like activity; therefore, the HRP-mimicking DNA enzyme (HRPzyme) catalyses the peroxidation in the presence of luminol and hydrogen peroxide. As a result of resonance energy transfer between luminol (donor) and dabcyl (acceptor), the chemiluminescence signals are extinguished in proportion to the concentration of OTA. The linear measurement range appeared to be within the range of 0.1–100 ng/mL, while the LOD was 0.22 ng/mL. The recovery levels of OTA for spiked coffee samples were 71.5% and 93.3%.

For the rapid detection of AOH, Apt-based optical waveguide aptasensors were developed. These sensors displayed an unprecedented LOD of 10.85 ± 0.77 fg/mL 1.55 ± 0.16 fg/mL and 0.52 ± 0.26 fg/mL, while the measuring range was found to be between 2.58 fg/mL and 25.82 ng/mL of AOH. Using the aptasensor, the detection of spiked wheat powder could be achieved with a LOD of 37 pg/g. The sensor was capable of 35 regenerations of 2 min each, and the test time, including the extraction of AOH from wheat, was only about 1 h [117].

Sun et al. [118] developed a direct aptasensor based on SPR for the detection of AFB1. The Apt was fixed on the sensor chip surface, and the SPR signal increased upon AFB1 binding. The aptasensor displayed a linear signal in the AFB1 concentration range between 0.13 ng/mL and 62.46 ng/mL, and had a LOD of 0.13 ng/mL. AFB1 has also been determined in food samples such as diluted red wine and beer.

Bianco et al. [119] developed a label-free, simple, and reliable Apt-based SPR sensor platform for the detection of OTA. Phase-interrogator SPR has proven to be a reasonably sensitive technique with a great utility in portable devices. The ssDNA Apt was immobilised using the mixed SAM immobilisation procedure in the presence of mercaptoundecanoic acid on the sensor surface of the SPR based on sinusoidal gratings. Under the optimised experimental conditions, the biosensor can detect 0.2 ng/mL OTA with a LOD of 5 pg/mL.

Nabok et al. [120] developed an Au nanostructure-based planar waveguide polarisation interferometer aptasensor for the detection of AFB1 allowing a detection range of 0.01–100 ng/mL. The sensor was further developed into a SiO_2_–Si_3_N_4_–SiO_2_ optical planar waveguide setup allowed detection of AFB1 at 1 pg/mL [121].

Wang et al. [122] achieved improved detection using a shortened Apt in a circular dichroism-based label-free aptasensor for AFB1. An improvement of over two orders of magnitude in the LOD resulted from the rational truncation of their AFB1-specific Apt, resulting in an LOD of 187 pg/mL.

In SERS-based aptasensors, the preparation of high-performance SERS tags is of key importance. Although Raman reporter molecules that are immobilised on the surface of noble metals are considered as sensitive SERS tags, external factors can negatively affect them, resulting in a reduced signal probe. Therefore, developing a SERS platform with good specificity, sensitivity, and stability is highly demanded. Embedding Raman reporters into core-shell NPs or in nanospheres can protect them from external factors which results in highly stable elevated signals.

An ultrasensitive SERS-based aptasensor for AFB1 determination was reported by He et al. [123], where Fe_3_O_4_@Au nanoflowers functionalised with cDNA were used as a capture probe and 4-mercaptobenzoic acid as a Raman reporter embedded in Au@Ag nanospheres functionalised with Cy3-modified AFB1 Apt as a reporter probe. As the affinity of the Apt is higher to AFB1 than that of cDNA, in the presence of AFB1, the reporter probe is released from the nanoflowers; therefore, SERS intensity is decreased and is inversely proportional to the concentration of AFB1. The sensor exhibited a wide linear range between 0.0001 and 100 ng/mL and a very low LOD of 0.4 pg/mL. By embedding the Raman reporter, the sensor showed excellent stability, sensitivity, and reproducibility.

Similarly, in the work of Chen et al. [124], Apt-modified Au@Ag core-shell NPs were used as a reporter probe and cDNA-modified gold nanorods as a capture probe in their sensor set-up for simultaneous determination of ZON and OTA in wheat and corn samples. For the determination of OTA, a linear measuring range of 0.01–100 ng/mL with a LOD of 18 pg/mL was achieved, while that of the determination of ZON was between 0.05 and 500 ng/mL, with a LOD of 0.054 ng/mL. In another experiment, cDNA-modified Fe_3_O_4_@Au was used as a capture probe for ZON determination. Using this sensor set-up, a linear range from 0.005 to 500 ng/mL was obtained with a LOD of 0.001 ng/mL [125].

A SERS-based aptasensor for multiplex determination of mycotoxins was reported by Song et al. [126]. In their set-up, silica photonic crystal microspheres (SPCMs) decorated with AuNPs functionalised with Apts were used, while AuNPs modified with corresponding anti-Apt sequences were applied to form SERS nanotags using Nile blue A and 5,5′-dithiobis (2-nitrobenzoic acid) dyes. With the optimised sensor, the AFB1 and OTA content of lily, jobstears seed, and lotus seed were determined. The dynamic measuring range was between 0.01 and 100 ng/mL for AFB1 and 0.001 and 10 ng/mL for OTA, with LODs of 0.36 pg/mL for AFB1 and 0.034 pg/mL for OTA.

Gillibert et al. [127] developed a highly sensitive and specific SERS sensor for the detection of OTA, using a rough gold film as a substrate, on the surface of which a specific Apt used as a bioreceptor was fixed. When the analyte was added, spectral differences appeared as a result of the interaction between the analyte and the specific Apt. Using the partial least squares regression method, the sensor can detect the concentration of OTA down to the picomolar range, which is much lower than the minimum concentration allowed in food. The linear measurement range was up to 0.40 µg/mL for OTA.

Single-mode detection methods are mainly used for the determination of mycotoxins. Their disadvantage is that these protocols are burdened with high interference. This can be mitigated by dual-mode detection, where false-negative results can be reduced by taking advantage of the two modes. He et al. [128] presented an aptasensor for FB1 determination using a dual-detection method: SERS and fluorometry. In their sensor set-up, a dual-mode nanoprobe was fabricated which consisted of gold nanorods modified with cDNA and the Apt of FB1 modified with Cy5.5. In the absence of FB1, the Apt and its cDNA associated, so the distance between the Au nanorod (AuNR) and the Cy5.5 was very small; therefore, strong SERS signals could be detected, while the fluorescent signal was weak. In the presence of FB1, the complex dissociated as the affinity of the Apt is higher to FB1 than to cDNA, resulting in decreased SERS signals and increased fluorescent signals. The SERS signals decreased linearly in the concentration range of 10–500 pg/mL, while the fluorescent signals increased linearly in a concentration range of 10–250 pg/mL. With the optimised sensor, FB1 concentrations in spiked wheat samples were determined.

Similarly, a dual-mode aptasensor was developed for the determination of OTA in coffee and wine samples, using OTA Apt-modified Au nanostars as a capture probe and Cy3-modified cDNA-functionalised gold nanospheres as a signal probe. The sensor had a low LOD of 0.17 ng/mL and 1.03 pg/mL in fluorescent and SERS mode, respectively [129].

An impedimetric aptasensor for the detection of OTA was developed based on a novel modifier-coated gold electrode consisting of electropolymerised Neutral Red and a mixture of AuNPs suspended in the dendrimer polymer Botlorn H30^®^ [130]. The OTA-specific thiolated Apt was covalently attached to the AuNPs via Au-S bonding. The aptasensor enabled the detection of 0.04–40.38 ng/mL 0.1–100 nM OTA (LOD: 0.01 ng/mL) in the presence of OTB in at least a 50-fold excess. The applicability of the aptasensor for real sample testing was confirmed using spiked beer samples. The recovery of 2 nM OTA was 70% for light beer and 78% for dark beer.

Han et al. [131] studied co-reduced molybdenum disulfide and AuNPs, and used them for the first time as an efficient platform that provided excellent electron transfer rates for EC electrodes and large surface area and solid connectivity for various Apts. After further modification with thionine and 6-(ferrocenyl)hexanethiol, a platform was achieved that allowed sensitive, selective, and simultaneous determination of two important mycotoxins, ZON and FB1. The aptasensor showed an excellent linear relationship for ZON and FB1 in the concentration range of 0.001–10 ng/mL and 0.001–100 ng/mL. The LOD was 0.5 pg/mL both for ZON and FB1, and the sensor showed excellent selectivity and stability. The efficiency of the aptasensor was verified on corn samples, and a satisfactory recovery was achieved.

Nucleic acid-based synthetic receptors, Apts, have been successfully selected for mycotoxins with high binding affinity and selectivity, and have been incorporated into many sensor platforms. Based on the optical properties of metallic NPs, aptamer-NPs assays were developed in various formats including solution- and paper-based set-ups that enabled cheap, fast, and sensitive testing for the detection of mycotoxins in contaminated food [136].

Wang et al. [132] developed an improved MB method for the rapid detection of AFB1, where a MB consisting of a DNA Apt flanked by carboxyfluorescein and a diazene-type fluorescence quencher using the complementary DNA (cDNA) strand, showed a higher fluorescence response to AFB1. After optimisation of key experimental factors, this method achieved rapid detection of AFB1 in the concentration range from 0.31 ng/mL to 0.94 μg/mL within 20 min, with a LOD of 0.31 ng/mL. The proper operation of the method was verified by testing diluted beer.

Qian et al. [133] developed a new, versatile aptasensor class for the specific detection of AFB1 using a two-channel detection method. AuNPs with peroxidase-like activity and promoting silver deposition have been used as versatile labels for both colourimetric and EC detection; therefore, Apt-modified Fe_3_O_4_@Au magnetic beads and cDNA-modified AuNP were developed as capture probes and signal probes. Through the hybridisation reaction between Apt and cDNA, a magnetic bead-Apt/cDNA-AuNPs bioconjugation was created. During the measurement, due to the high affinity between the Apt and AFB1, the cDNA-AuNPs detached from the magnetic bead-Apts. The released signal probes were separated and collected using an external magnetic field and directed to both colourimetric and EC detection channels, allowing AFB1 detection in the concentration ranges of 5–200 ng/mL and 0.05–100 ng/mL, respectively. The corresponding LODs were 35 pg/mL and 0.43 pg/mL, and applicable in corn samples.

A new type of computer simulation test was introduced to design new and highly functional Apt probes for the detection of mycotoxins. Mousivand et al. [134] used two new AFB1-binding Apts as recognition elements in lateral flow aptasensors and a reflective phantom interface (RPI) platform. A new variant was obtained using a previous one designed using a genetic algorithm-based in silico maturation strategy, through a truncation scheme and computational simulation approach. Based on the designed probes, two Apt-AuNP strip biosensors were developed for the competitive detection of AFB1. In the LFA method designed, the novel and the original Apts showed IC_50_ values of 2.9 and 15.4 ng/mL, and a dynamic measurement range of 0.1–50 and 0.5–50 ng/mL, respectively. Based on the estimated LOD, the starting Apt (0.1 ng/mL) was more sensitive compared to its truncated form (0.5 ng/mL). Based on their in silico and experimental selectivity against other mycotoxins, both test strips proved to be selective for AFB1. Both developed aptasensors were successfully used to detect AFB1 in corn flour within 30 min using a simple strip reader.

Jiang et al. [135] developed a novel dual-mode aptamer sensor for the detection of AOH using a ferrocenecarboxylic acid-DNA_2_ quenching electrochemiluminescence (ECL) and electrochemical (EC) signal response probe. Ru-MOF/Cu@Au NPs were used as an ECL substrate platform to detect AOH via a competitive reaction between AOH and ferrocenecarboxylic acid-DNA_2_. The advantage of the dual-mode aptamer sensor is the rapid synthesis based on electrodeposition of Ru-MOF on the electrode surface and, since the bifunctional reagent ferrocenecarboxylic acid is conjugated to the Apt, it detects both ECL and EC signals, increasing the accuracy of the result. The determination of AOH showed a measurement range of 100 fg/mL to 100 ng/mL, with LODs of 14–83 fg/mL. The suitability of the sensor was proven by testing spiked fruit samples.

## 5. Peptides Used for Sensoric Application

Peptides are natural or synthetic short chains of amino acids. Their chemical structure is identical to the specific sequence found in proteins, but at the same time they show high stability, easily modified structure, and considerable chemical versatility, enabling the replacement of antibodies as bioreceptors for the selective binding of small molecules for the design of biosensors [137,138]. The specific peptide ligands used for the analysis can be designed at a relatively low cost with the help of computational modelling programs [139]. Based on the second-generation peptide library, phage-type peptides with higher binding affinity to target compounds can be produced. A dodecapeptide mimotope was isolated for the determination of OTA using the aforementioned second-generation peptide library. In the case of the chemiluminescent enzyme-linked immunosorbent system developed using a mimotope, the IC_50_ and the linear measurement range were 0.04 ng/mL and 0.006–0.245 ng/mL, respectively [140]. However, only a few peptides have been successfully used as biorecognition elements, as the limited knowledge of the interactions involved in molecular recognition is a difficulty when designing new peptide receptors with high affinity.

## 6. Conclusions and Future Perspectives

From the aspect of food and feed safety, preventing mycotoxin contamination is of utmost importance. However, contamination is often unavoidable due to unfavourable weather conditions or improper storage practices. Therefore, rapid analytical control and detection methods can play a crucial role in ensuring food safety. As evident from this review, numerous biosensors with different structures have been developed in the past decades for the detection of mycotoxins. The advancements in using new biorecognition units, such as Abs, nanobodies, peptides, Apts, and MIPs/nanoMIPs provide a solid foundation for the determination of mycotoxins using different detection systems.

**Figure 3:**
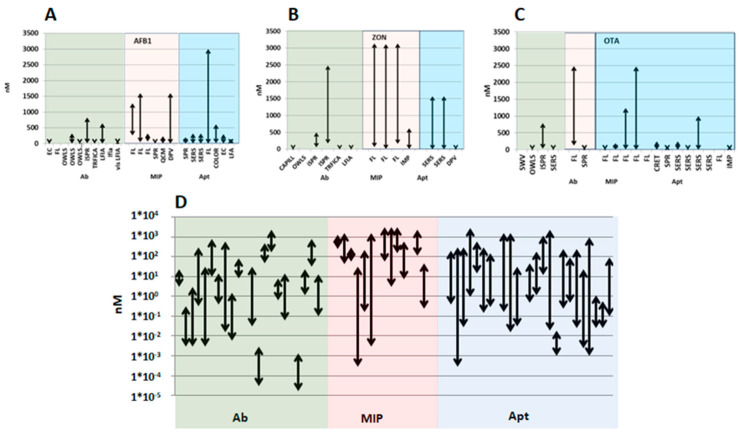
Detection ranges of various biosensor applications. Biosensors based on antibodies (Ab), molecularly imprinted polymers (MIP), and aptamers (Apt) for the detection of aflatoxin B1 (AFB1) (**A**), zearalenone (ZON) (**B**), and ochratoxin A (OTA) (**C**), and a summary diagram grouped according to biosensitive compounds on log scale (**D**).

Based on the articles summarised in this review, we compared the measurement ranges of Ab-, MIP-, and Apt-based sensors used for AFB1, ZON, and OTA analysis (Figure 3). Although the detection method differs for each measurement, the conclusion can be drawn that, in the case of measurements with Abs, it is usually possible to measure in a lower and narrower concentration range, often even at pM–nM concentrations. Researchers typically achieved the lowest sensitivity using MIPs, although there are exceptions. The available measurement range when using MIPs was in the nM-μM range. Based on the results, the widest measurement range can be obtained using Apts, providing, in many cases, comprehensive measurement possibilities with the sensors in the pM–μM range. The advantage of the aptamer biosensor is its high sensitivity, good selectivity, wide detection range, and low LOD, making it a promising candidate for the development of further biosensors [117].

Regarding the detectors, there are also significant differences in sensitivity. Label-free techniques typically offer very low LODs and high sensitivity, whereas faster methods, such as LFIA, may only achieve significantly lower sensitivity. However, it is important to note that the measurement limits of these devices correspond well to the threshold values established for food safety, and therefore, a reasonably accurate assessment of the contamination in the given sample is provided. It should be noted that, for some articles, the LOD value is higher than the lower limit of the measurement range, which is due to the way the LOD is calculated (S/N = 3).

Based on the recent literature and food safety requirements, it is predicted that multiple mycotoxin detection systems will become more prominent. Mahmoudpour et al. [141] also mention the applicability of SPR sensors. Moreover, to address the specificity limitations of the biorecognition units, new developments are needed which incorporate novel types of bioreceptors into SPR biosensors. These advancements are expected to enhance the sensitivity of mycotoxin determination while mitigating issues related to contamination and matrix effects. It is also worth noting, however, that these biosensors currently find limited practical use and are found primarily in laboratory tests and clinical diagnostics. Therefore, the commercialisation of the developments in mycotoxin detection remains an immense challenge.

In summary, since mycotoxin pollution poses an increasing challenge to both food raw material producers and food safety experts, two main directions are expected to emerge for the development of biosensors: primarily, techniques suitable for on-site control (e.g., LFA sticks) and high-sensitivity, multiple mycotoxin determination procedures suitable for laboratory control. Among the biologically sensitive units, the use of Apt is becoming more and more widespread, even if its selectivity is often lower than that of Ab applications. The use of nanomaterials continues to play a major role in the construction of various biosensors, as they significantly improve the sensitivity of individual sensors. Among the detectors, electrochemical coupled techniques (e.g., ECL) may represent the direction of further development, as well as techniques suitable for the determination of the already mentioned multiple mycotoxins, such as iSPR.

## Figures and Tables

**Table 1:** Comparison of monoclonal and polyclonal antibodies.

Feature	Antibodies	
	Monoclonal	Polyclonal
Specificity	monospecific	polyspecific
Product standardisation	easy	varies from batch to batch
Cross-reactivity	low	high
Production	expensive	less expensive
Concentration of non-specific IgG	low	high

**Table 2:** A comprehensive list of antibody-based biosensors against various mycotoxins.

Mycotoxin	Analytical Method	Linear Range	Limit of Detection	Samples	Reference
PAT	EIS, GCE/GO-anti-PAT	0.01–10 ng/mL (0.065–64.9 nM)	9.8 pg/mL (64 pM)	apple juice	[44]
OTA	polarisation interferometer		10 pg/mL (25 pM)		[45]
OTA	SWV/BSA/anti-OTA mAb/Mal- polyethylene glycol-NH_2_/MPA/AuNps/GCE	7.17–548.8 fg/mL (0.018–1.36 pM)	2.04 fg/mL (0.005 pM)	spiked beer, corn	[46]
AFB1	polarisation interferometer		10 pg/mL (32 pM)		[47]
AFB1	EC, rGO-Ni NPs/anti-AFB1/ITO	1–8 ng/mL (3.20–25.6 nM)	0.16 ng/mL (0.51 nM)		[48]
AFB1 DON	fluorescent probes, UCNPs (NaYF4:Yb/Ho/Gd/antibody-target complex	1–100 pg/mL (3.2–320 pM); 1–100 pg/mL (3.4–340 pM)	1 ng/mL (3.2 pM); 1 pg/mL (3.4 pM)	adulterated peanut oil	[49]
ZON	competitive capillary- immunofl/glass capillary/ZON-BSA	0.01–10 ng/mL (0.031–31.4 nM)	3 fg/mL (9.4 fM)		[50]
ZON	polarisation interferometer/polyelectrolyte/protein A		10 pg/mL (31 pM)		[51]
ZON	OWLS/ZON-conj./pAb	0.01–1 pg/mL (0.031–3.14 pM)	2 fg/mL (6.3 fM)	maize	[52]
AFB1 OTA	OWLS/Ag-conj./mAb	0.001–1 ng/mL (0.0032–3.20 nM); 0.5–10 ng/mL (1.24–24.8 nM)	0.5 pg/mL (1.6 pM); 0.1 ng/mL (6.3 pM)	wheat, barley	[53]
AFB1	OWLS/AFB1-conj./mAb	0.1–100 ng/mL (0.32–320 nM)		spice paprika	[54]
AFB1	OWLS/AuNPs/AFB1-conj./mAb	0.001–10 ng/mL (0.0032–32 nM)		spice paprika	[55]
DON		0.005–50 ng/mL (0.0169–168.7 nM)	1 pg/mL (3.38 pM)	wheat flour	[56]
FB1 DON	EC, ITO/AuNPs/anti-FB1; anti-DON	0.3–140 ng/mL (0.42–194 nM); 0.2–60 ng/mL (0.6875–202.5 nM)	97 pg/mL (0.134 nM); 35 pg/mL (0.118 nM)	corn	[57]
AFB1 OTA ZON DON	SPR/SAM/Ag-conj.		0.59 ng/mL (0.189 nM); 1.27 ng/mL (3.145 nM); 7.07 ng/mL (22.21 nM); 3.26 ng/mL (11.0 nM)	corn, wheat	[58]
DON ZON T-2 OTA FB1 AFB1	iSPR 6-plex Ag-OVA conj./mAbs	26–3200 µg/kg (0.088–10.8 μM); 16–160 µg/kg (0.050–0.503 μM); 0.6–290 µg/kg (1.3–622 nM); 13–320 µg/kg (0.032–0.792 μM); 10–1200 µg/kg (0.14–1.66 μM); 3–260 µg/kg (10–833 nM)	26 μg/kg (88 nM); 6 μg/kg (18 nM); 0.6 μg/kg (1.3 nM); 3 μg/kg (7.4 nM); 2 μg/kg (2.8 nM); 0.6 μg/kg (2 nM)	barley	[59]
DON ZON T-2	iSPR/Ag; AuNPs- secondary Ab- conj.	48–2827 ng/g (0.16–9.54 μM); 54–790 ng/g (0.17–2.48 μM); 42–1836 ng/g (0.09–3.94 μM)	15 µg/kg (50 nM); 24 µg/kg (80 nM); 12 µg/kg (30 nM);	wheat	[8]
AFM1	UV/TRFM–ICTS (Eu(III)-TRFM/mAb)	0.05–2.0 ng/mL (0.15–6.09 nM)	19 pg/mL (57 pM)	milk and its products	[60]
AFB1 ZON	TRFICA/Eu/Tb(III)/AIdNb)/mAb	0.13–4.54 ng/mL (0.42–14.54 nM); 0.20–2.77 ng/mL (0.63–8.70 nM)	50 pg/mL (160 pM); 70 ng/mL (220 pM)	corn and its products	[61]
DON, ZON	iSPR/mycotoxin–protein		21 ng/mL (70.9 nM); 17 ng/mL (57.4 nM); 16 ng/mL (50.3 nM); 10 ng/mL (31.4 nM)	corn, wheat	[62]
FB1 AFB1	LFIA/mAb/Fe-N-C SAzyme	0.02–150 ng/mL (0.028–207.80 nM); 0.005–200 ng/mL (0.016–640.5 nM)	13.9 pg/mL (19.3 pM); 2.8 pg/mL (9 pM)		[63]
AFB1 ZON FB1 DON OTA T-2	multiplex SERS-based LFIA in./Raman reporter molecules/Au@Ag/mAbNPs/Ag-conj.	2–500 pg/mL (0.006–1.60 nM); 0.02–5 ng/mL (0.063–15.705 nM); 0.5–35 ng/mL (0.69–48.49 nM); 0.2–10 ng/mL (0.69–48.49 nM); 0.05–5 ng/mL (0.124–12.382 nM); 0.05–5 ng/mL (0.107–10.72 nM)	0.96 pg/mL (3.1 pM); 6.2 pg/mL (19 pM); 0.26 ng/mL (360 pM); 0.11 ng/mL (371 pM); 157 pg/mL (389 pM); 8.6 pg/mL (18.4 pM)	maize	[64]
AFB1	visual LFIA/Ab-GO	0.3–1 ng/mL (0.96–3.20 nM)	0.3 ng/mL (0.96 nM)	peanut oil, maize, rice	[65]
DON	rapid LFIA/anti-DON mAbs/FMs	2.5–25 ng/mL (8.44–84.4 nM)	2.5 ng/mL (8.44 nM)	agricultural products	[66]
TeA	visual LFIA/AuNP or AuNF/anti-TeA McAb	12.5–100 ng/mL (63.4–507 nM); 0.78–50 ng/mL (3.96–253.5 nM)	12.5 ng/mL (63.4 nM); 0.78 ng/mL (3.95 nM)	apple juice, tomato ketchup	[22]

**Table 3:** A comprehensive list of molecularly imprinted polymer-based biosensors against various mycotoxins.

Mycotoxin	Analytical Method	Linear Range	Limit of Detection	Samples	Reference
AFB1	FL/CdTe QDs/MIPs	80–400 ng/g (0.26–1.28 μM)			[76]
AF	FL/Mn-ZnS QDs/MIP		16 ng/mL (50 nM)	almond-, soy-, rice-based beverages	[77]
STC	FL/luminescent CD-MIP	0.05–2 µg/mL (0.15–6.17 μM)	0.19 µg/mL (0.59 nM)	millet, maize, rice	[78]
STC	FL/carbon nanosheets MIP	0.049–1.0 µg/mL (0.15 to 3.1 μM)	24 ng/mL (74 nM)		[79]
ZON	FL/CQDs-MIP	0.02–1.0 µg/mL (0.06–3.14 μM)	0.02 µg/mL (0.06 μM)	maize	[80]
ZON	FL/MIOM/CdSe/ZnS QDs	0.96–993 ng/mL (0.003–3.12 μM)	0.64 ng/mL (2 nM)	corn, rice, wheat flour	[81]
ZON	FL/MOF/MIP	0.05–1.0 µg/mL (0.16–3.14 μM)	18 ng/mL (56.5 nM)	wheat	[82]
PAT	FL/AgNPs/Zn-MOF	0.015–1.54 μg/mL (0.1–10 μM)	9.24 ng/mL (60 nM)	surface water, apple juice	[83]
OTA	FL/silica-UCNPs/MIP	0.05–1 µg/mL (0.12–2.48 μM)	31 ng/mL (76.8 nM)	corn, rice, feed	[84]
AFB1	FL/MIP	14–500 ng/mL (0.045–1.60 μM)	14 ng/mL (45 nM)	waste water	[85]
AFB1	smartphone-based FL MIP	20–100 ng/mL (0.06–0.32 μM)	20 ng/mL (64 nM)	maize	[86]
CIT	Disp. FL/fiber optic/MIP	0.5–2.5 μg/mL (2.0–10.0 μM)			[87]
TeA	luminescent sensor/Eu(III)/MIP	1.7–20 μg/mL (8.6–101.4 μM)	0.5 μg/mL (2.5 μM)	rice	[24]
TeA	luminescent/SiO_2_@Ru-MIP	0.10–78.9 μg/mL (0.51–400 μM)	63.8 ng/mL (323 nM)		[88]
OTA	SPR/MIP	0.1–20 ng/mL (0.25–49.5 nM)	0.028 ng/mL (70 pM)	dried fig	[89]
AFB1	SPR/MIP/AuNPs	0.0001–10 ng/mL (0.0003–32 nM)	1.04 pg/mL (3.3 pM)	corn, peanut	[90]
AFM1	SPR/MIP/AuNPs	0.0003–20 ng/mL (0.0009–60.92 nM)	0.4 pg/mL (1.2 pM)	milk	[91]
DON	SPR/MIP	0.1–100 ng/mL (0.34–337 nM)	1 ng/mL (>3.37 nM)		[92]
CIT	SPR/MIP	0.005–1.0 ng/mL (0.02–4.0 nM)	1.7 pg/mL (6.8 pM)	red yeast rice	[93]
PAT	SERS/AuNPs/MIP	0.00108–7.7 ng/mL (0.007–50 nM)	0.83 pg/mL (5.37 pM)	fruit product	[94]
PAT	QCM/MIP	7.5–60 ng/mL (48.7–389.3 nM)	3.1 ng/mL (20.1 nM)	apple and pear juice, haw flakes	[95]
AFB1	QCM/AuNPs/MIP	0.05–75 ng/mL (0.16–240.17 nM)	2.8 pg/mL (9 pM)	peanut, pistachio, rice, wheat	[96]
CIT	QCM/AuNPs/MIP	1.5–50.1 ng/mL (6.0–200 nM)	0.45 ng/mL (1.8 nM)	cereal	[97]
PAT	DPV/GCE/QDs/AuNPs@Cu-MOF/MIP	0.001–70.0 ng/mL (0.0064–454.2 nM)	0.7 pg/mL (4.5 pM)	apple juices	[98]
PAT	DPV/Au@PANI/SeS2@Co MOF/SPE	0.000154–15.4 ng/mL (0.001–100 nM)	0.102 pg/mL (0.66 pM)	apple juice	[99]
PAT	DPV/MIP-Au/CS-CDs/GCE	0.000154–0.15 ng/mL (0.001–1 nM)	0.117 pg/mL (0.757 pM)	fruit juices	[100]
PAT	EC/poly(thionine/graphene/PtNPs/MIP	0.002–2 ng/mL (0.013–12.98 nM)	1 pg/mL (6.5 pM)	apple and grape juice	[101]
AFB1 FB1	DPV/ITO/PANI-MIP	0.001–500 ng/mL (0.003–1601 nM); 0.001–500 ng/mL (0.0014–692.7 nM)	0.31 pg/mL (1 pM); 0.32 pg/mL (4.4 pM)	corn	[102]
FB1	DPV/PtE/PP-Zn-porphyrin MIP	0.72–7220 fg/mL (0.001–10 pM)	0.02 fg/mL (0.03 fM)	maize	[18]
ZON	Impedimetric sensor/SCAuE/PPD-MIP	2.5–200 ng/mL (7.9–628 nM)	0.20 ng/mL (0.628 nM)	corn flakes	[103]
T-2	DPV/Fe^3+^-MIP-GCE	0.51–0.99 ng/mL (0.0011–2.12 μM)	0.15 ng/mL (0.33 nM)	cereals, human serum	[16]
CIT	PVP/SPE/GF/MIP	25–12,500 ng/mL (0.1–5.0 μM)	5 ng/mL (19 nM)	red rice, cranberry, turmeric, corn, wheat germ, rice starch	[21]
CIT	voltammetric/GCE/PtNP/rGO	0.25–25 fg/mL (1–100 pM)	0.05 fg/mL (0.2 pM)	rye	[104]
CIT	DPV/MIP/PdNPs/BZ/GQDs/GCE	250–1250 fg/mL (1–5 nM)	50 fg/mL (0.2 nM)	chicken egg	[105]

**Table 4:** A comprehensive list of aptamer-based biosensors against various mycotoxins.

Mycotoxin	Analytical Method	Linear Range	Limit of Detection	Samples	Reference
OTA	total internal reflection ellipsometry		10 pg/mL (25 pM)		[109]
OTA	label-free FL/CdTe QDs/Apt/(N-methyl-4-pyridy) porphyrin (TMPyP)	0.2–20 ng/mL (0.49–49.5 nM)	0.16 ng/mL (0.40 nM)	*Astragalus membranaceus*	[110]
OTA	FL/ZnCdSe QDs/self-assembled Zn porphyrin	0.5–80 ng/mL (1.24–198 nM)	0.33 ng/mL (0.82 nM)	coffee, milk	[111]
OTA	label-free FL/SWCNHs	5–500 ng/mL (12.4–1240 nM)	2.3 ng/mL (5.70 nM)	red wine	[112]
OTA	FL/MB-fluorophore/free-cDNA	10 pg/mL–1 µg/mL (0.02–2480 nM)	0.247 pg/mL (0.61 pM)	wheat	[113]
OTA	FL/dendritic/DNA/ AMNPs/OTA-Apt	0.4–8.1 pg/mL (1–20 pM)	0.04 pg/mL 0.10 pM	corn	[114]
PAT	FRET/rare-earth-doped UCNPs/Apt	0.01–100 ng/mL (0.06–648.90 nM)	3 pg/mL (20 pM)	apple juice	[115]
OTA	CRET/Apt	0.1–100 ng/mL (0.25–248 nM)	0.22 ng/mL (0.55 nM)	coffee	[116]
AOH	FL/optical-fibre waveguide/Apt	2.6 fg/mL–25.8 ng/mL (10 fM–100 nM)	10.85 fg/mL (42 fM)	wheat	[117]
AFB1	SPR/Apt	0.13–62.46 ng/mL (0.4–200 nM)	0.13 ng/mL (0.4 nM)	red wine, beer	[118]
OTA	SPR/Apt	0.2–40 ng/mL (0.50–99.1 nM)	5 pg/mL (12.4 pM)		[119]
AFB1	polarisation interferometer/Au nanostructure-or SiO_2_–Si_3_N_4_–SiO_2_ waffle-based		1-10 pg/mL (2.5–25 pM)		[120,121]
AFB1	label-free circular dichroism/Apt		187 pg/mL (0.6 nM)		[122]
AFB1	SERS/Fe_3_O_4_@Au NFs-cDNA/Au-4MBA@Ag NSs-Apt	0.0001–100 ng/mL (0.0003–320 nM)	0.4 pg/mL (1.3 pM)	peanut oil	[123]
ZON OTA	SERS/Au NRs-cDNA/Au@4-MBA@Ag CS-OTA-Apt or Au@DTNB@Ag CS-ZEN-Apt	0.05–500 ng/mL (0.16–1570 nM); 0.01–100 ng/mL (0.025–248 nM)	0.054 ng/mL (0.17 nM); 0.018 ng/mL (0.045 nM)	wheat, corn	[124]
ZON	SERS/Au@DTNB@Ag CS-Apt/Fe_3_O_4_@Au MNPs-cDNA	0.005–500 ng/mL (0.016–1570.50 nM)	0.001 ng/mL (0.003 nM)	beer, wine	[125]
AFB1 OTA	SERS/SPCM/AuNPs- Apts/AuNPs-anti-Apt-dyes	0.01–100 ng/mL (0.03–320.23 nM); 0.001–10 ng/mL (0.0025–24.76 nM)	0.36 pg/mL (0.001 nM); 0.034 pg/mL (0.084 pM)	lily, jobstears seed, lotus seed	[126]
OTA	SERS/rough gold film/Apt	0.40 pg/mL–0.40 µg/mL (1 pM–1 μM)			[127]
FB1	SERS and FL/cDNA-AuNR/Apt-Cy5.5	10–500 pg/mL (0.014–0.69 nM); 10–250 pg/mL (0.014–0.35 nM)	3 pg/mL (0.0042 nM); 5 pg/mL (0.0069 nM)	wheat	[128]
OTA	SERS and FL/Au nanostars-cDNA-Apt	10–500 pg/mL (0.025–1.24 nM); 10–250 pg/mL (0.025–0.62 nM)	1.03 pg/mL (0.0026 nM); 0.17 pg/mL (0.4 pM)	coffee, wine	[129]
OTA	impedimetric/Au electrode/electropolymerised Neutral Red-AuNPs-Apt/Botlorn H30^®^	0.04–40.38 ng/mL (0.1–100 nM)	0.01 ng/mL (0.02 nM)	beer	[130]
ZON FB1	DPV/GCE/rMoS2-Au/APs/BSA/L-CPs	0.001–10 ng/mL (0.031–31.41 nM); 0.001–100 ng/mL (0.031–314.10 nM)	0.5 pg/mL (1.6 pM)	corn	[131]
AFB1	FL/cDNA/MB/Apt	0.31 ng/mL–0.94 μg/mL (1 nM–3 μM)	0.31 ng/mL (1 nM)	beer	[132]
AFB1	colourimetric and EC/Apt-Fe_3_O_4_@Au magnetic beads/Apt/cDNA-AuNPs	5–200 ng/mL (15.23–609.24 nM); 0.05–100 ng/mL (0.15–304.62 nM)	35 pg/mL (0.11 nM); 0.43 pg/mL (0.0013 nM)	corn	[133]
AFB1	LF A/Apt-AuNP strip	0.1–50 ng/mL (0.30–152.3 nM)	0.1 ng/mL (0.30 nM)	corn	[134]
AOH	ECL or EC/GCE by Ru-MOF/Cu@Au NPs/ferrocenecarboxylic acid-DNA_2_	0.1 pg/mL–100 ng/mL (0.4 pM–387.25 nM)	0.014 pg/mL (0.054 pM); 0.083 pg/mL (0.32 pM)	apples, oranges, pears	[135]

## Data Availability

Not applicable.

## Abbreviations

Ab: antibody; AF: aflatoxin; AFB1: aflatoxin B1; AFB2: aflatoxin B2; AFG1: aflatoxin G1; AFG2: aflatoxin G2; AFM1: aflatoxin M1; AFM2: aflatoxin M2; AgNP: silver nanoparticle; AIdNb: anti-idiotypic nanobody; AMNPs: amino-modified magnetic nanoparticles; AOH: alternariol; Apt: aptamer; AuNP: gold nanoparticle; AuNR: gold nanorods; BSA: bovine serum albumin; CD: carbon dots; CIT: citrinin; CRET: chemiluminescence resonance energy transfer; DON: deoxynivalenol; DPV: differential pulse voltammetry; EC: electrochemical; EIS: electrochemical impedance spectroscopy; ELISA: enzyme-linked immunosorbent assay; FB1: fumonisin B1; FM: fumonisin; FRET: fluorescence resonance energy transfer; GCE: glassy carbon electrode; GF: graphene nanoflakes; GO: graphene oxide; HPLC: high-performance liquid chromatography; HPLC-MS: high-performance liquid chromatography with mass spectrometric detection; HRP: horseradish peroxidase; HRPzyme: HRP-mimicking DNA enzyme; IC_50_: mean (50%) inhibitory concentration; iSPR: imaging surface plasmon resonance; ITO: indium tin oxide; LFA: lateral flow analysis; LFIA: lateral flow immunoassay; LOD: limit of detection; LOQ: limit of quantification; mAb: monoclonal antibody; MB: molecular beacon; MIOM: molecularly imprinted optosensing material; MIP: molecularly imprinted polymer; MOF: metal-organic framework; NP: nanoparticle; OT: ochratoxin; OTA: ochratoxin A; OTB: ochratoxin B; OVA: ovalbumin; OWLS: optical waveguide lightmode spectroscopy; pAb: polyclonal antibody; PAT: patulin; PtNP: platinum nanoparticle; QCM: quartz crystal microbalance; QD: quantum dot; SAzyme: single-atom nanozyme; SERS: surface-enhanced Raman scattering; SPCM: silica photonic crystal microsphere; SPR: surface plasmon resonance; STC: sterigmatocystin; SWV: square-wave voltammetric; SWCNHs: single-walled carbon nanohorns; T-2: T-2 toxin; TRFICA: time-resolved fluorescence immunochromatographic assay; TRFM: time-resolved fluorescent microsphere; TRFM-ICTS: time-resolved fluorescent microsphere immunochromatographic test strip; UCNP: up-conversion nanoparticle; ZON: zearalenone.
